# Fault Diagnosis Method of Plunger Pump Based on Meta-Learning and Improved Multi-Channel Convolutional Neural Network Under Small Sample Condition

**DOI:** 10.3390/s25154587

**Published:** 2025-07-24

**Authors:** Xiwang Yang, Jiancheng Ma, Hongjun Hu, Jinying Huang, Licheng Jing

**Affiliations:** 1 School of Information and Communication Engineering, Shanxi University of Electronic Science and Technology, Linfen 041000, China; yangxw@nuc.edu.cn; 2 School of Computer Science and Technology, North University of China, Taiyuan 030051, China; b1907076@st.nuc.edu.cn (J.M.); s202107016@nuc.edu.cn (H.H.); 3 School of Mechanical Engineering, North University of China, Taiyuan 030051, China; s202202021@st.nuc.edu.cn

**Keywords:** fault diagnosis, plunger pump, small sample, meta-learning

## Abstract

A fault diagnosis method based on meta-learning and an improved multi-channel convolutional neural network (MAML-MCCNN-ISENet) was proposed to solve the problems of insufficient feature extraction and low fault type identification accuracy of vibration signals at small sample sizes. The signal is first preprocessed using adaptive chirp mode decomposition (ACMD) methods. A multi-channel input structure is then employed to process the multidimensional signal information after preprocessing. The improved squeeze and excitation networks (ISENets) have been enhanced to concurrently enhance the network’s adaptive perception of the significance of each channel feature. On this basis, a meta-learning strategy is introduced, the learning process of model initialization parameters is improved, the network is optimized by a multi-task learning mechanism, and the initial parameters of the diagnosis model are adaptively adjusted, so that the model can quickly adapt to new fault diagnosis tasks on limited datasets. Then, the overfitting problem under small sample conditions is alleviated, and the accuracy and robustness of fault identification are improved. Finally, the performance of the model is verified on the experimental data of the fault diagnosis of the laboratory plunger pump and the vibration dataset of the centrifugal pump of the Saint Longoval Institute of Engineering and Technology. The results show that the diagnostic accuracy of the proposed method for various diagnostic tasks can reach more than 90% on small samples.

## 1. Introduction

As the key power component of rotary machinery equipment, plunger pumps are widely used in various types of industrial equipment. After a long time conducting high-intensity work, the internal parts of the plunger pump will become worn or damaged, resulting in frequent failures during the operation of the equipment. Therefore, condition monitoring and fault diagnosis for the plunger pump can guarantee the normal operation of industrial equipment, reduce the frequency of trouble-free shutdown, and improve the working efficiency and safety of the equipment [1].

Due to the complex internal structure of the plunger pump, the harsh working environment, and processing errors, the fault modes are varied and difficult to diagnose accurately. In the early days, fault diagnosis of the plunger pump was mainly based on the observations and judgment of experienced technicians on the site and was carried out, for example, by listening to whether the plunger pump had abnormal sound during operation and observing whether there was oil leakage. However, with the rapid progress of sensing technology, detection technology, and information technology, with the help of various sensors installed in the key parts of the plunger pump, a large number of detailed operating parameters and status information can be collected in real time, and then these data can be used for deep fault identification and diagnosis. Intelligent fault diagnosis methods based on deep learning have gradually become the focus of current research [2]. By constructing multi-level and highly abstract fault diagnosis models and developing targeted learning algorithms with excellent performance, these methods can accurately mine the internal distribution characteristics and evolution rules of fault data from the complex data. Thus, the key characteristic information reflecting the essence of equipment fault can be extracted efficiently. Chao et al. [3] fused the vibration data of the three channels of the plunger pump, classified them using a convolutional neural network (CNN), and achieved good results. Eraliev, O et al. [4] proposed a stackable convolutional autoencoder (SCAE) that can extract more hierarchical features from vibration signals. Experimental results verified the effectiveness of SCAE in the fault diagnosis of small-sample plunger pumps. Tang et al. [5] converted plunger pump vibration signals into images using continuous wavelet transform (CWT) and then classified them by CNN with high classification accuracy. Based on reference [5], Tang et al. [6] used the Bayesian optimization (BO) algorithm to carry out the adaptive learning of hyperparameters for a CNN and proposed an improved CNN (CNN-BO) to further improve the classification accuracy. Zhu et al. [7] established an adaptive convolutional neural network model to classify five typical working conditions of the plunger pump. Zhu et al. [8] used synchronous compression transform (SWT) to divide plunger pump signals into two dimensions, time and frequency, and achieved intelligent plunger pump fault diagnosis by using the established Visual Geometry Group long short-term memory (VGG-LSTM) model. He et al. [9] proposed a deep multi-signal fusion adversarial model (MFAN) based on transfer learning to address the problems of diverse working conditions and inconsistent data distribution presented by the plunger pump. A multi-signal fusion module was designed to assign weight to vibration signals and sound signals, and MFAN showed good performance in cross-domain fault diagnosis of the plunger pump. However, traditional deep learning models cannot distinguish which features are more important for the task at hand. Xu et al. [10] combined the channel attention mechanism squeeze-and-excitation network (SENet) with a one-dimensional convolutional neural network. SENet could adjust the weights of different input channels but did not take into account the dynamic adaptation of thresholds.

The continuous maturation and innovation of deep learning technology greatly promotes the development of technology in the field of fault diagnosis. However, the training of deep neural networks requires a large amount of fault sample data with complete label information. In engineering practice, in order to ensure the safety and continuous operation of equipment, equipment cannot be run in a faulty state for a long time during the entire operation cycle. Most of the data obtained during the operation cycle is unlabeled and in a healthy state, while only part of the data records the characteristic information of the equipment failure. Therefore, it is necessary to study how to maximize the limited fault sample information to improve the accuracy and effectiveness of fault diagnosis models [11]. The proposal of meta-learning has improved these problems to some extent [12]. The core idea of meta-learning is to learn the general knowledge of multiple learning tasks, which provides favorable prior knowledge for solving new tasks and ultimately helps to improve the generalization ability of the model. Model agnostic meta-learning (MAML) [13] and other meta-learning techniques train the model under a variety of different learning tasks so that it can adapt to new learning tasks with only a small number of training samples. This method has achieved good performance in computer vision [14], speech recognition [15], and reinforcement learning [16]. In the past few years, a series of studies have been conducted to explore the application of meta-learning in the field of fault diagnosis. Lin et al. [17] proposed generalized model-agnostic meta-learning (GMAML) for fault diagnosis driven by heterogeneous signals, which was verified by bearing vibration signals and sound signals. Luo et al. [18] proposed a meta-learning method for bearing signals under variable speed conditions. Based on the elastic prototype network, the designed enhanced feature encoder and elastic measurer were used to complete cross-domain fault diagnosis under variable speed conditions. Liu et al. [19] proposed a class-incremental continual learning model for plunger pump faults based on weight space meta-representation (WSMR) continuous learning model for the class incremental fault diagnosis of plunger pumps, aiming at the problem of DL models suffering catastrophic forgetting during continuous learning, and used modified WaveletKernelNet (MWKN) to reduce the forgetting of old knowledge. Li et al. [20] proposed an attention-based deep meta transfer learning method (ADMTL) to address problems such as poor model generalization ability in low-frequency fine-grained fault diagnosis tasks. ADMTL introduced an attentional mechanism to guide the learning of feature learners. It can be seen from the above studies that domestic and foreign scholars have made progress in the fault diagnosis field by using meta-learning technology, successfully developing a variety of intelligent fault diagnosis technologies driven by small sample data, providing new ideas for meta-learning in small-sample fault diagnosis direction. However, traditional meta-learning strategies do not have a task-specific parameter selection strategy. We consider the degree of difference between different tasks and the unique attributes of common faults of plunger pumps, and further research is conducted under the condition of data limitation to build a meta-learning model for small-sample plunger pump fault diagnosis, solving the problem of data scarcity while improving the model’s rapid adaptability and generalization performance when facing unknown plunger pump fault modes.

In this paper, a fault diagnosis method of plunger pump based on meta-learning and improved multi-channel convolutional neural network (MAML-MCCNN-ISENet) is proposed, which introduces MAML theory into the established MCCNN-ISENet deep learning model. The main contributions are as follows:

(1) In this paper, a soft threshold structure is added to the structure of SENet, and ISENet is proposed, which uses the weight distribution characteristics learned by each channel under the attention mechanism to automatically generate the corresponding optimal threshold setting, which enhances the overall recognition accuracy and stability.
(2) In this paper, in order to solve the problem of insufficient generalization ability of the model in the small-sample scenario, the MAML model is improved by combining the meta-learning strategy, the learning process of the model initialization parameters is optimized, and the improved MAML is combined with the deep learning model to improve the rapid adaptability and generalization performance of the model in the face of unknown plunger pump failure modes.
(3) In this paper, the proposed method is verified on the plunger pump fault dataset and the centrifugal pump vibration dataset of the San Longoval Institute of Engineering and Technology, and the accuracy of the proposed method is higher than that of several small-sample fault diagnosis methods.

The rest of this paper is organized as follows. Section 2 illustrates the related work. Section 3 describes the proposed MAML-MCCNN-ISENet in detail. Section 4 validates the effectiveness of the MAML-MCCNN-ISENet method with two datasets. The conclusions and future research directions are illustrated in Section 5.

## 2. Related Work

### 2.1. Multi-Channel Convolutional Neural Network (MCCNN)

#### 2.1.1. Multi-Channel One-Dimensional Convolution

In deep learning and machine learning, the multichannel input layer is a fundamental and widely used component. This structure enhances the processing and interpretation of diverse datasets, improving prediction and recognition performance in complex scenarios. The multi-channel input layer relies on manually engineered sample features, which characterize samples from multiple perspectives and dimensions—analogous to “observing” the same sample through different lenses. These multi-angle representations are fed into distinct channels of the feature map, enabling the model to learn comprehensive sample features. The fused feature map thus captures the sample’s essential information, facilitating effective prediction and recognition.

Multi-channel one-dimensional convolution [21,22] is a deep learning algorithm designed for time series data processing. Its key characteristic lies in employing multiple parallel one-dimensional convolutional kernels, each dedicated to extracting features from distinct input channels. The input typically consists of one-dimensional time series data, making this method particularly effective for signal fusion. By integrating time series signals from multiple sources or perspectives, it achieves more comprehensive feature representations, thereby enhancing model performance and generalization capability in time series analysis tasks.

#### 2.1.2. Structure of Multi-Channel Convolutional Neural Network

The basic structure of an MCCNN is as follows:

(1) Input: The input of the network is a multi-channel one-dimensional signal form, and the signals of different channels can be similar information or different types of data.
(2) One-dimensional convolution: The data in each channel are independently carried out one-dimensional convolution operations to obtain the feature information of each channel with local relevance.
(3) Pooling: Reduce the scale of the feature map and extract more abstract and important features.
(4) Full connection layer: The extracted local features of each channel are mapped to the same high-dimensional space, and the feature information of all channels is deeply fused through full connection operation.

In MCCNN, the convolutional layer extracts local features through linear weighted combinations of input data yet lacks nonlinear processing capability. To address this limitation, an activation layer typically follows each convolutional layer, transforming the linear outputs into nonlinear representations. This sequential architecture enables the neural network to better capture complex data patterns and features through nonlinear mapping.

Following the activation layer, the network typically undergoes pooling to reduce feature dimensionality and mitigate overfitting, thereby enhancing model generalization. The pooling layer serves dual purposes: (1) decreasing computational complexity and parameter count in subsequent layers, while (2) preserving and refining locally salient features. This operation ultimately improves the model’s generalization performance on unseen data.

Through successive processing by convolutional, activation, and pooling layers, the network effectively extracts and filters the primary features from the input data. Upon completion of the final pooling operation, the resulting feature map contains highly informative representations. The fully connected layer then flattens this output into a one-dimensional feature vector for subsequent classification or regression tasks.

The MCCNN architecture enables parallel processing of multi-channel input signals while effectively fusing extracted channel-specific information. This design achieves complementary integration of cross-channel features, maximizing information utilization to enhance both depth and breadth of data representation. By leveraging multi-dimensional components through parallel convolution operations, the model overcomes single-channel limitations, leading to improved classification performance and accuracy.

### 2.2. Channel Attention Mechanism (CAM)

**Figure 1:**
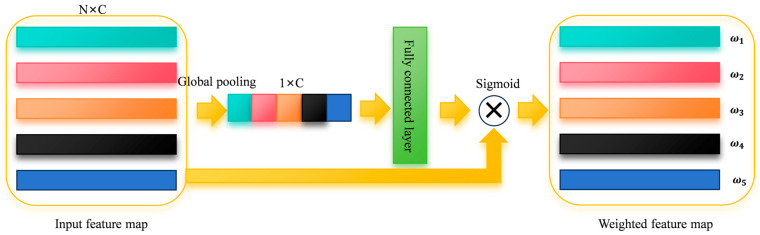
Channel attention mechanism.

The channel attention mechanism (CAM) [23] enhances convolutional neural networks by adaptively weighting channel-wise features. As illustrated in Figure 1, this approach extends successful attention applications from image and natural language processing domains. These mechanisms enable networks to dynamically prioritize informative input components. Since feature importance varies across channels—with some containing critical information and others potentially comprising noise—CAM strategically focuses network resources on the most salient channels. This selective emphasis improves feature representation while suppressing irrelevant information.

#### Squeeze and Excite Networks (SENet)

The Squeeze and Excitation Network (SENet) [24] introduces a novel attention-based learning paradigm. This architecture recognizes that distinct feature channels contribute differentially to classification performance across varying samples. SENet employs a compact subnetwork to learn channel-specific weighting coefficients, which are then multiplied with corresponding feature channels to adaptively recalibrate their relative importance. This mechanism effectively implements dynamic channel-wise attention allocation within the network.

**Figure 2:**
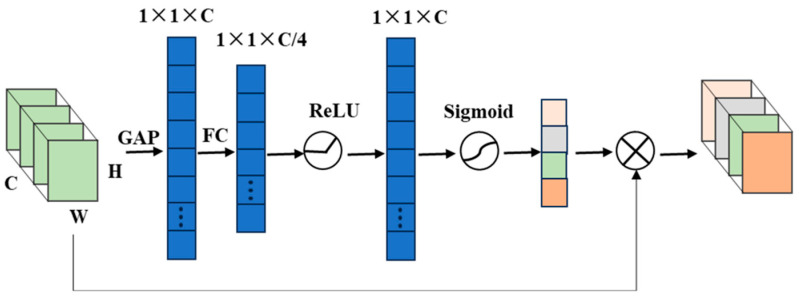
SENet networks.

In SENet, the module first extracts global channel information through global average pooling (GAP) of input features, enabling effective fault information extraction. The architecture then employs two fully connected (FC) layers to establish and analyze inter-channel correlations, with matching input-output dimensions to generate channel-specific weighting coefficients. These coefficients undergo Sigmoid normalization, constraining values to the [0,1] range to quantitatively represent each channel’s relative importance. Finally, a scale operation performs channel-wise multiplication, applying the normalized weights to corresponding features. This selective enhancement mechanism allows the SE module to emphasize the most impactful channels for model performance. As illustrated in Figure 2, the module processes inputs with dimensions C × H × W, where C, H, and W denote channel count, signal height, and signal width, respectively.

### 2.3. Meta-Learning

#### 2.3.1. Basic Theory of Meta-Learning

Meta-learning, as an advanced machine learning paradigm, acquires transferable learning principles through systematic analysis of multiple task experiences. This approach enables rapid adaptation to novel tasks by leveraging accumulated knowledge. The framework synergistically combines the robustness of statistical learning with the representational capacity of deep neural networks. Such integration addresses two critical challenges: (1) preventing overfitting in data-scarce scenarios while (2) fully exploiting deep learning’s pattern recognition capabilities. Consequently, meta-learning enhances both learning efficiency and cross-task generalization performance through optimized knowledge transfer.

The meta-learning framework enables models to extract highly discriminative feature representations from severely limited fault samples, while simultaneously optimizing classifier architecture for improved accuracy. This dual capability ensures reliable fault type identification and localization under data scarcity conditions. Through meta-learning principles, the diagnostic model achieves robust performance even in data-deficient scenarios, demonstrating particular effectiveness for small-sample fault diagnosis applications.

Meta-learning operates through two fundamental mechanisms: First, it enables machines to “learn how to learn” by assimilating problem-solving experiences. This equips the system with both analytical capabilities and the adaptive capacity to derive novel solutions for unfamiliar problems by synthesizing past knowledge with current requirements. Second, it facilitates superior knowledge transfer, allowing models to not only generalize learned patterns to new tasks but also creatively adapt and integrate this knowledge to address substantially different problem domains.

In practical meta-learning architectures, the system typically employs a dual-component framework consisting of base learners and a meta-learner. Each base learner specializes in task-specific learning and optimization, while the meta-learner operates at a higher abstraction level. Through systematic observation and analysis of multiple base learners’ performance across diverse tasks, the meta-learner extracts transferable knowledge to guide subsequent task learning. The architecture implements an iterative refinement process: upon completion of each task-specific training, base learners transmit acquired knowledge to the meta-learner, which subsequently updates its strategic parameters. This cyclic interaction progressively enhances the initialization states and learning rules provided to base learners for new tasks, ultimately improving convergence speed and overall system performance.

#### 2.3.2. Model Agnostic Meta-Learning (MAML)

**Figure 3:**
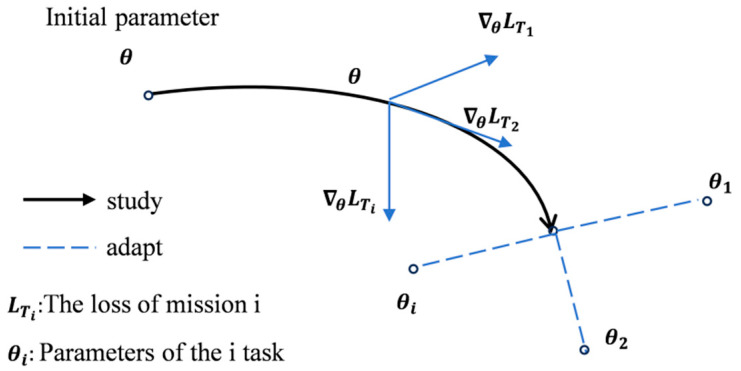
MAML parameter optimization process.

Proposed by Chelsea Finn at UC Berkeley in 2017, the Model-Agnostic Meta-Learning (MAML) algorithm represents a highly versatile approach within meta-learning research. As illustrated in Figure 3, MAML operates through two distinct phases: meta-learning (outer loop) and task adaptation (inner loop). The algorithm’s architecture enables rapid adaptation to novel tasks through its unique bi-level optimization process. The inner loop employs task-specific learning, where any base network model undergoes training using task-specific data to generate corresponding parameters. These parameters subsequently compute task-specific loss functions, which are optimized via backpropagation. Meanwhile, the outer loop functions as a meta-learner, systematically aggregating knowledge across tasks to optimize the model’s initialization parameters through iterative updates. Key advantages of MAML include the following:

Model-agnostic flexibility, allowing application across diverse machine learning architectures; efficient knowledge transfer through gradient-based meta-optimization [25]; and rapid convergence on new tasks with limited samples via learned initialization parameters. This dual-loop mechanism enables the model to acquire universally adaptable initialization parameters, facilitating quick adaptation to unfamiliar tasks while maintaining strong generalization performance. The framework’s effectiveness stems from its ability to integrate prior knowledge from multiple tasks into fundamental parameter settings, establishing an optimal starting point for new task learning.

To obtain this set of initial parameters, MAML’s optimization problem is expressed as

(1) $$\theta \prime =\arg\min_{\theta }\left[\sum_{T_{i}\sim P(\tau )}L_{T_{i}}(f_{\theta_{i}^{\prime }})=\sum_{T_{i}\sim P(\tau )}L_{T_{i}}f(\theta -\alpha \nabla_{\theta }L_{i}(f_{\theta }))\right]$$

where $\theta -\alpha \nabla_{\theta }L_{i}(f_{\theta })$ is the gradient updating process of MAML inner loop parameters, and α is the inner loop learning rate, $\nabla_{\theta }L_{i}(f_{\theta })$ is the gradient of the loss function $L_{i}(f_{\theta })$ to the parameter θ. $T_{i}$ represents task i; P(τ) is the distribution of task sets. In the outer loop, the meta-parameter update process is

(2) $$\theta =\theta -\beta \nabla_{\theta }\sum_{T_{i}\sim P(\tau )}L_{T_{i}}(f_{\theta_{i}^{\prime }})$$

where β represents the learning rate of the meta-learner. By constantly updating the inner and outer ring parameter θ, the MAML algorithm can learn a better basic model parameter θ, and solve the problem of small sample learning through several fine-tuning.

## 3. The Proposed Method

### 3.1. Improved SENet Model (ISENet)

The structure of SENet is enhanced in this paper by incorporating a soft threshold mechanism, the soft thresholding operation, originally proposed by Donoho and Johnstone [26] for wavelet denoising, Zhang et al. [27] used a combination of soft threshold operations and residual networks to suppress noise interference, we introduced soft thresholds into SENet in order to dynamically adjust the thresholds for individual channels based on their learned weight distribution features under the attention mechanism. This enables the model to emphasize important features while suppressing minor or noisy ones, thereby significantly improving key feature retention and resistance to noise interference during information screening. As a result, overall recognition accuracy and stability are enhanced.

Soft threshold is the core step of many noise reduction methods, which sets the feature value whose absolute value is below a certain threshold τ to zero, and shrinks the feature whose absolute value is greater than that threshold to zero. The function of soft threshold is expressed as follows:

(3) $$y=\left\{\begin{array}{ll} x-\tau , & x>\tau \\ 0, & 0\leq x\leq \tau \end{array}\right.$$

where τ is the threshold, *x* and *y* are the input and output features, respectively. As can be seen from the above formula, soft thresholding can actually set the feature value to zero in any interval, which is a more flexible way to eliminate the feature in a specific range.

In previous studies [26], the threshold value is determined by experience. This paper makes the threshold more targeted by improving the SE module to automatically set the threshold value for each feature channel.

**Figure 4:**
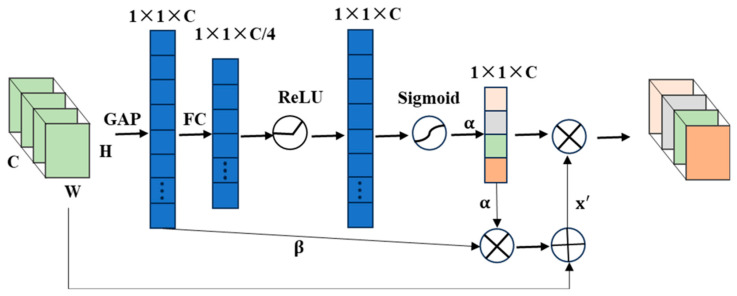
Improved SENet.

Figure 4 shows the improved SENet with an extra layer of soft-threshold output compared with the previous SENet. The weight α obtained by the attention mechanism and the absolute eigenvalues obtained on the *C* channel are averaged to obtain the eigenvalue β. Therefore, the threshold on each channel is set to τ=α⋅β, and then the input on each channel is soft-threshold. To obtain output $x^{\prime }$, in order to make full use of the weight distribution obtained by attention, improve network performance, and enhance the weight related to classification, the output $x^{\prime }$ and weight α are focused on the feature channel.

The enhanced SENet architecture incorporates an innovative feature-weighting mechanism that dynamically adjusts channel-wise thresholds during classification. This adaptive approach provides two key advantages over the conventional structure: (1) sample-specific threshold determination for precise noise suppression, and (2) automated learning of cross-channel dependencies to eliminate feature redundancy. These modifications collectively improve the network’s discriminative feature extraction capability while maintaining computational efficiency.

### 3.2. Multi-Channel Convolutional Neural Networks Incorporating Attention Mechanisms (MCCNN-ISENet)

**Figure 5:**
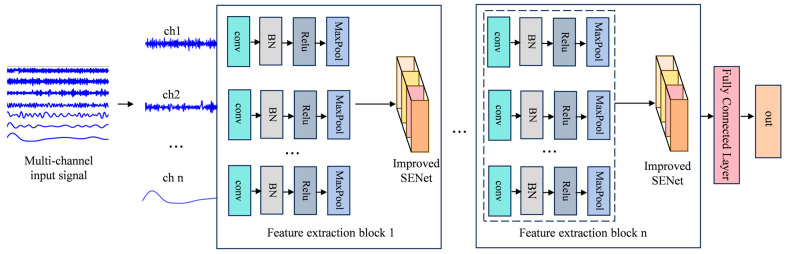
Overall network structure.

The proposed MCCNN-ISENet architecture integrates advanced attention mechanisms to enhance feature learning from multi-channel input signals, as illustrated in Figure 5 with detailed layer configurations provided in Table 1. The network employs sequential convolutional blocks for hierarchical feature extraction, where each block contains a 1D convolutional layer followed by batch normalization to stabilize training by reducing internal covariate shift. The ReLU activation function introduces nonlinear transformations while maintaining computational efficiency, and subsequent max-pooling layers progressively reduce spatial dimensions to expand receptive fields and improve feature robustness. A key innovation of this architecture lies in its channel interaction encoder, which combines enhanced SENet attention mechanisms with convolutional operations to model cross-channel dependencies and extract discriminative diagnostic features. This integrated approach dynamically adjusts feature representations based on inter-channel relationships while preserving important spatial patterns through the convolutional pathway. The normalization layers accelerate convergence and improve training stability by standardizing activations throughout the network, while the pooling operations optimize computational efficiency without sacrificing critical feature information. The architecture’s dual-path design effectively balances local feature extraction through convolutional processing with global channel relationship modeling via attention mechanisms, resulting in improved diagnostic performance across varying operating conditions. By jointly optimizing these complementary operations during end-to-end training, the network learns robust representations that capture both detailed fault characteristics and their contextual relationships within the multi-channel signal space.

### 3.3. Improvement of the MAML Algorithm

The MAML framework aims to enable rapid model adaptation to new tasks by learning transferable initial parameters through multi-task training. By optimizing across diverse but related tasks, MAML obtains parameter initialization with strong cross-task generalization potential. To further improve performance, we integrate weight decay regularization and momentum-based optimization into the MAML update process, enhancing both generalization capacity and training stability. However, a key limitation persists: initialization parameters optimal for certain training tasks may not generalize effectively to novel tasks, potentially hindering rapid adaptation. This fundamental challenge necessitates developing more robust parameter initialization strategies that maintain adaptability across varying task distributions.

#### 3.3.1. Weight Decay (L2 Regularization)

Weight decay serves as an effective regularization method to mitigate model overfitting. This L2 regularization approach improves model generalization by constraining weight magnitudes through a penalty term during optimization. The technique operates by augmenting the standard gradient update with an additional parameter-dependent penalty, effectively balancing model complexity and performance. Mathematically, L2 regularization modifies the loss function through the addition of a squared weight penalty term, expressed as

(4) $$L_{\mathrm{total}}=L_{\mathrm{original}}+\frac{\lambda }{2}\sum_{i=1}^{m}\omega_{i}^{2}$$

where $L_{\mathrm{original}}$ is the original loss function, is the *i*-th weight parameter, which is the coefficient corresponding to each feature, λ is the regularization intensity (weight attenuation coefficient), which controls the influence of the regularization term, and *m* is the total number of model parameters.

#### 3.3.2. Momentum Term

The momentum technique accelerates gradient-based optimization by incorporating historical gradient information to guide parameter updates. This approach combines current gradient computations with exponentially decaying averages of past gradients, effectively reducing oscillations while maintaining consistent descent directions. By introducing a velocity term that accumulates gradient momentum, the method achieves faster convergence through smoother parameter trajectories and improved escape from local optima. The resulting update rule modifies standard gradient descent through momentum-based adjustments.

(5) $$\nu_{t}=\gamma \nu_{t-1}+\eta \nabla_{w}L_{t}$$

(6) $$w_{t}=w_{t-1}-v_{t}$$

where $\nu_{t}$ is the momentum variable at time step, γ is the momentum factor (momentum term coefficient), η is the learning rate, and $\nabla_{w}L_{t}$ is the gradient of the loss function L with respect to the parameter w at time step t.

#### 3.3.3. MAML Parameter Update and Optimization

The MAML algorithm facilitates few-shot learning on novel tasks, yet its limited training samples often induce overfitting, ultimately degrading meta-testing performance. To address this, we integrate L2 regularization into MAML’s optimization framework, effectively constraining model complexity while improving feature discriminability. Furthermore, the incorporation of momentum term accelerates inner-loop convergence, particularly in optimization landscapes with steep gradients or high curvature, thereby generating more stable and reliable parameter updates.

Here is a combination of MAML’s two-layer update logic, taking into account L2 regularization and the parameter update mode of the momentum term:

In the MAML outer loop update, the gradient of the loss function after adaptation to task is first calculated:

(7) $$g_{\theta^{\prime }}^{\left(i\right)}=\nabla \theta^{\prime }L_{T_{i}}(f_{\theta_{T_{i}}^{\prime }})+\lambda \theta_{T_{i}}^{\prime }$$

where $\theta_{T_{i}}^{\prime }$ is the parameter updated after an inner loop on task $T_{i}$, and λ is the L2 regularization coefficient.

When updating the inner loop, the momentum term is introduced, and the momentum vector m is first initialized as zero or a small constant, and then updated as follows:

(1) Calculate the loss gradient of the current task (to account for regularization)

(8) $$g_{t}=\nabla_{\theta^{\prime }}L_{T_{i}}(f_{\theta_{t}^{\prime }})+\lambda \theta_{t}^{\prime }$$

(2) Update the momentum vector according to Equation (5):

(9) $$v_{t}=\gamma v_{t-1}+\alpha g_{t}$$

(3) Parameters in the MAML algorithm are combined with the momentum term:

(10) $$\theta_{t+1}^{\prime }=\theta_{t}^{\prime }-v_{t}$$

Repeat the above internal cycle updating steps until the adaptation is complete and obtain the new parameter $\theta_{T_{i}}^{\prime }$ after adaptation. Finally, the adaptive task loss gradient update element parameter θ is used in the outer loop:

(11) $$\theta \leftarrow \theta -\beta \sum_{T_{i}}g_{\theta^{\prime }}^{\left(i\right)}$$

Here, β is the outer cycle learning rate, γ is the momentum factor, and α is the inner cycle learning rate. Through this dual updating mechanism, the MAML model can not only use L2 regularization to prevent overfitting when adapting to new tasks but also use momentum term to accelerate convergence and provide a more stable learning process.

#### 3.3.4. Parameter Initialization Based on Specific Tasks

The standard MAML implementation employs identical initialization parameters across all tasks, failing to account for their individual characteristics. This limitation can be addressed by developing task-specific initial parameters to reduce the adaptation loss for new tasks [28]. In conventional MAML, the parameter space becomes constrained once the initial weights and network architecture are fixed, as each task’s parameters are strictly derived from previous model iterations. To overcome this constraint, we propose an enhanced parameter selection strategy that incorporates task-relevant initialization parameters, thereby improving MAML’s adaptation effectiveness.

Specifically, after each iteration, the output features obtained by the forward propagation training of the initial parameters and the gradient parameters obtained by the back propagation of each task are saved. In order to learn the features and losses of different tasks as much as possible, the following formula is constructed:

For a model with *n* tasks,

(12) $$F=\left[\max(F_{1}),\ldots ,\max(F_{n})\right]$$

(13) $$L=\left[mean(\nabla L_{1}),\ldots ,mean(\nabla L_{n})\right]$$

where $F_{i}$ is the output value obtained for each task using the current parameter, thus obtaining the feature mapping and gradient of each task under the current parameter. To fuse the extracted parameters into the network, build a fully connected structure:

(14) F+L→Linear+ReLU→[…]Linear+Sigmoid→ωi

**Figure 6:**
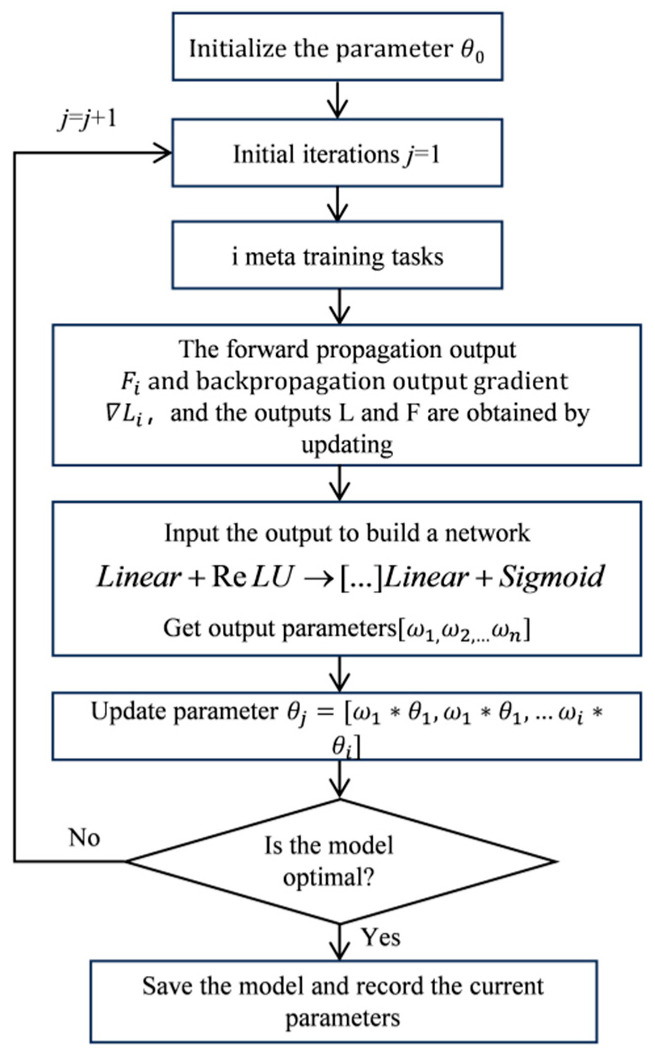
Parameter update flow chart.

The *F*, *L*, obtained above are concatenated as the input of the fully connected network, and then nonlinear activation is carried out through ReLU. After a full connection, the mapping $\omega_{i}$ of each task is obtained through Sigmoid nonlinear activation, and the weighting of each task is realized. Figure 6 shows the update process diagram.

### 3.4. Fault Diagnosis Process of Plunger Pump Based on MAML-MCCNN-ISENet

To address plunger pump fault diagnosis under small-sample conditions, this paper presents an improved MAML-based diagnostic approach. The method employs an MCCNN-ISENet architecture as the core diagnostic model within the meta-learning framework. Through strategic integration of multi-task learning mechanisms, the optimized network achieves rapid adaptation to new tasks with limited samples while maintaining generalization performance. This design effectively mitigates overfitting risks and significantly improves diagnostic accuracy in data-scarce scenarios.

The MAML-MCCNN-ISENet plunger pump diagnostic framework operates through two sequential phases: meta-training and meta-testing. The meta-training phase employs diverse fault diagnosis tasks to optimize the MCCNN-ISENet model globally, yielding a robust parameter initialization that demonstrates strong cross-task generalization. These pre-trained parameters enable rapid adaptation to novel fault conditions, achieving convergence with minimal task-specific data during the meta-testing phase.

**Figure 7:**
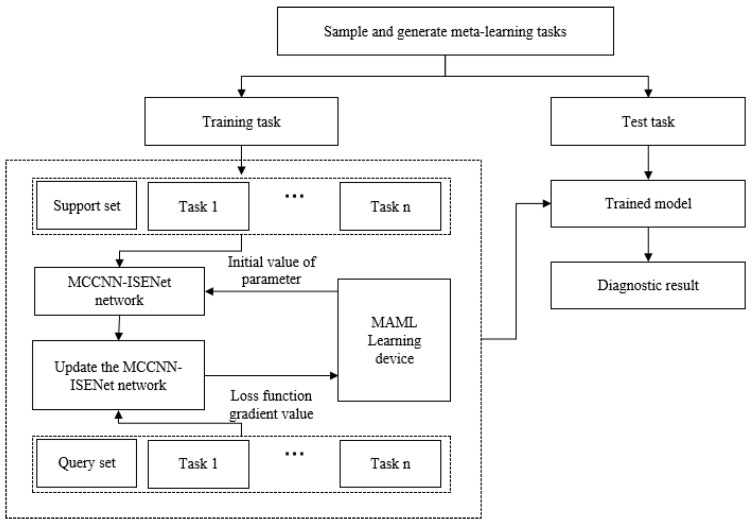
Flow chart of model training and testing.

During meta-testing, the base model undergoes fine-tuning using the pre-trained initialization parameters from meta-training, enabling rapid adaptation to new fault diagnosis tasks. With only minimal training samples required for parameter updates, the model efficiently optimizes its settings to improve both convergence speed and diagnostic precision for previously unseen fault patterns. The complete workflow of the MAML-MCCNN-ISENet approach for plunger pump fault diagnosis is depicted in Figure 7.

**Figure 8:**
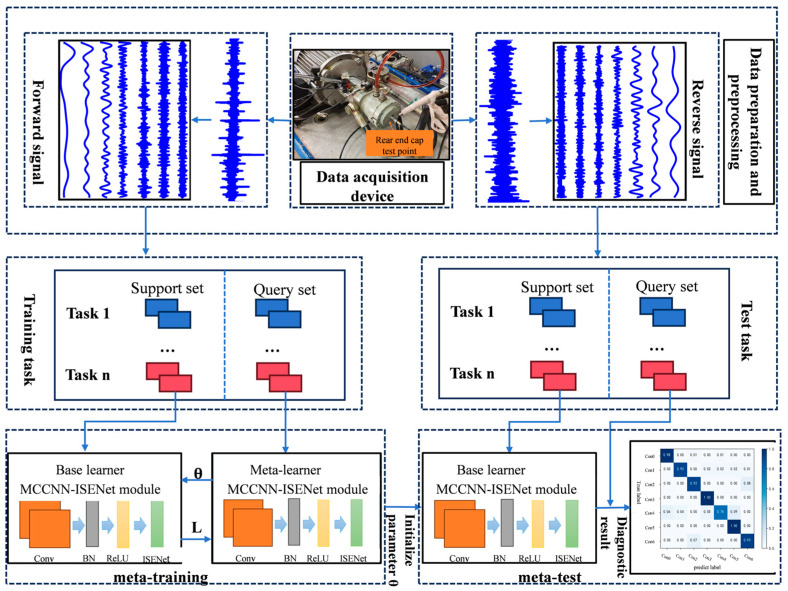
Overall diagnostic framework.

The entire fault diagnosis process of a plunger pump, based on MAML-MCCNN-SENet, is divided into the following stages: initial data acquisition, data preprocessing, dataset construction, task configuration, model training and testing. The overall framework is shown in Figure 8.

Step 1: Construct a fault diagnosis test platform for plunger pumps to acquire vibration signals under various operational conditions and fault modes.

Step 2: The collected raw vibration signals undergo preprocessing via Adaptive Chirp Mode Decomposition (ACMD) to extract multidimensional feature components characterizing the plunger pump’s health conditions.

Step 3: The processed data is partitioned into training and test sets, with random sampling to create support–query pairs. Following meta-learning principles, multiple diagnostic meta-tasks are constructed within the training set. Each meta-task contains a number of support set samples for the initial update of the model parameters, and an independent query set sample to evaluate the model’s generalization performance on new fault types.

Step 4: A MAML-optimized diagnostic model is constructed using the MCCNN-ISENet feature encoder. The model undergoes multi-level optimization on the training set, where the meta-learning algorithm identifies optimal initial parameters enabling rapid adaptation to new fault diagnosis tasks.

Step 5: For new operational conditions or unseen fault types in the test set, the meta-trained parameters serve as initialization values, enabling rapid task adaptation through efficient gradient updates. This approach yields precise fault diagnosis based on the optimized model’s performance.

## 4. Experimental Results

### 4.1. Case 1: Laboratory Plunger Pump Fault Diagnosis Dataset

#### 4.1.1. Experiment Description

**Figure 9:**
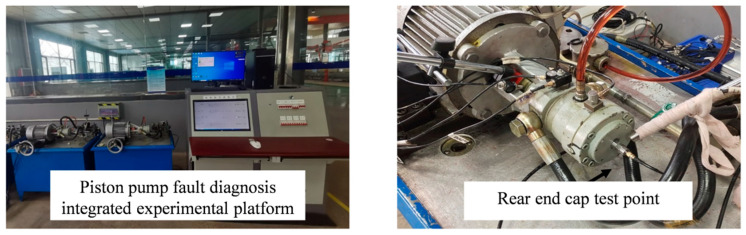
Test bench and test point diagram.

In this paper, the monitoring data of ZT type axial plunger pump fault diagnosis monitoring test bench for electro-hydraulic switch machine built by our research group were verified experimentally. Since vibration signals can often reflect the potential fault information inside the equipment, the vibration acceleration signals measured by the acceleration sensor installed on the rear end cover of the plunger pump were selected as the data. The experimental test bench and measuring point are shown in Figure 9.

**Figure 10:**
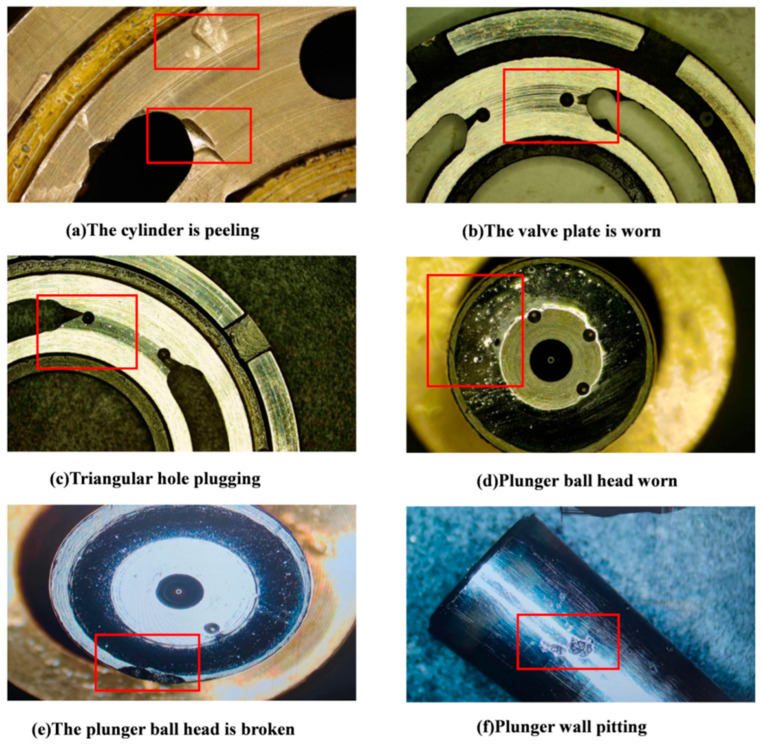
Plunger pump fault conditions: (**a**) the cylinder is peeling, (**b**) the valve plate is worn, (**c**) triangular hole plugging, (**d**) plunger ball head worn, (**e**) the plunger ball head is broken, (**f**) plunger wall pitting.

Under the standard working conditions of 960 r/min motor speed and 12 Mpa outlet pressure, the sampling frequency is set to 5120 Hz, and the vibration signals under forward transmission and reverse state are continuously collected for 15 min. The experimental subjects selected the plunger pump that failed in actual operation of the railway department for test bench monitoring. The fault samples included common fault types of plunger pump, including cylinder spalling, valve plate wear, plunger wall pitting, triangle hole blockage, plunger ball head fracture, plunger tail fracture, etc. The specific details of each fault are shown in Figure 10.

In this paper, a fault diagnosis test bench of the plunger pump is selected to collect data for verification, and the vibration acceleration signal of the measuring point of the rear end cover of the plunger pump is selected. The experimental data cover seven typical states of the plunger pump under the two working conditions of forward and reverse rotation, including normal state, cylinder spalling, valve plate wear, plunger wall pitting, triangle hole plugging, plunger ball head wear, and plunger ball head fracture. Each fault state is treated as an independent classification category, yielding seven labeled data subtypes. For comprehensive analysis, each fault type includes sample groups from both rotational directions, resulting in 14 representative sample sets. To ensure statistical reliability, five experimental trials were conducted, with final accuracy reported as the mean performance across all trials. The complete dataset is utilized for model training and evaluation, thoroughly validating the proposed method’s diagnostic effectiveness and accuracy for plunger pump fault detection.

Table 2 presents the constructed dataset configuration, where 20 consecutive and non-overlapping sample instances are selected for each fault category, with each sample comprising 2048 sequentially sampled data points. To validate the diagnostic model, we employ a rigorous evaluation protocol: first training on sub-dataset A (forward rotation condition) and subsequently testing on sub-dataset B (reverse rotation condition). The MAML implementation adopts an N-way K-shot learning paradigm, where each meta-task is constructed by (1) randomly selecting n categories from the seven available fault types in dataset A, and (2) sampling k signal instances per category to form a complete n × k classification task. This process is iterated to generate multiple diverse meta-tasks for comprehensive training. The same sampling strategy is applied to dataset B for testing, creating a robust framework for evaluating few-shot learning performance. This experimental design specifically enhances the model’s capability to rapidly adapt to novel fault conditions with limited samples.

#### 4.1.2. Analysis of Experimental Results

To rigorously evaluate the model’s diagnostic performance across various scenarios, we implemented a systematic experimental framework with multiple N-way K-shot configurations (N = 3, 5, 7; K = 1, 5, 10, 15). Based on preliminary accuracy and computational efficiency analyses, we selected the 3-way setting for detailed investigation. Each configuration maintained balanced support sets containing matched samples from three distinct plunger pump fault categories. The support set composition scaled with K values: 1-shot tasks contained one sample per fault, while 10-shot and 15-shot tasks incorporated 10 and 15 samples per category, respectively, ensuring consistent evaluation metrics across all experimental conditions.

To maintain experimental consistency, the query set for each task was proportionally configured to mirror its corresponding support set, containing matching numbers of fault samples (1, 5, 10, or 15 per category). This symmetrical design enables rigorous evaluation of the model’s few-shot fault recognition capability when guided by limited training examples.

A total of 200 such 3-way multilocal fault classification tasks were constructed throughout the training and testing process, which together constituted the supporting set dataset for training and adjusting model parameters during the internal iteration process. A series of key parameters set at the beginning of model training and their initial values are further listed in Table 3. The regularization coefficient λ in the L2 regularization term in MAML is searched between [1 × 10^−4^, 1 × 10^−3^], and finally λ is selected as 5 × 10^−4^, and the momentum factor γ in the momentum term in MAML is selected as 0.9 according to the literature [29].

**Figure 11:**
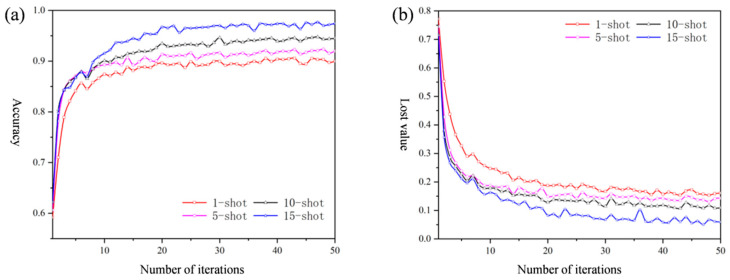
(**a**) Accuracy trend graph during model training. (**b**) Trend chart of loss value during model training.

During model training, the loss value and accuracy rate of the meta-training set under the classification tasks of 3-way 1-shot, 3-way 5-shot, 3-way 10-shot, and 3-way 15-shot are shown in Figure 11. It can be intuitively observed from the figure that after 30 iterations, the loss function value of the model gradually decreases and tends to converge, and the training trend of the model is generally the same for all four classification tasks.

The 3-way 15-shot configuration demonstrates superior performance, achieving both faster convergence and higher final accuracy compared to other settings. This enhancement stems from the increased sample availability, which enables more comprehensive learning of fault characteristics. The additional training samples facilitate (1) deeper pattern recognition of fault features, (2) more precise classification boundary determination, and (3) improved generalization to novel fault instances. Consequently, the model develops robust diagnostic capabilities that translate to significantly better performance in the 15-shot tasks.

Table 4 shows the classification accuracy of the various methods under the 3-way 1-shot, 5-shot, 10-shot, and 15-shot tasks. It can be observed that the accuracy of various models increases with the increase in fault samples for the model to learn. Especially under the condition that the number of fault signals increases to 15, the proposed model achieves the best classification accuracy of 97.29%, demonstrating its excellent adaptability and generalization ability in small sample learning problems.

**Figure 12:**
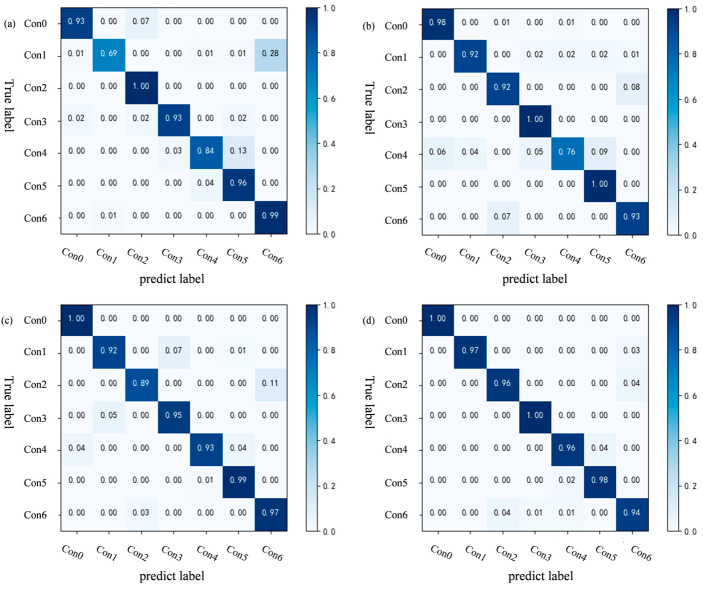
Confusion matrix of four classification tasks on the laboratory plunger pump fault diagnosis dataset (**a**) 3-way 1-shot task; (**b**) 3-way 5-shot task; (**c**) 3-way 10-shot task; (**d**) 3-way 15-shot task.

The confusion matrices presented in Figure 12 detail the classification performance of our model across various few-shot learning tasks. Notably, the model maintains robust diagnostic capability, achieving accuracy rates exceeding 69 percent for all fault types even in the most challenging one-shot learning scenario. This performance demonstrates significant improvement as sample availability increases, with fifteen-shot tasks attaining accuracy levels above 94 percent for every fault category. The model’s exceptional overall accuracy of 97.29 percent conclusively establishes its effectiveness and reliability for plunger pump fault diagnosis applications.

#### 4.1.3. Ablation Experiment Analysis

A series of ablation experiments were conducted to investigate the performance of the model and the effects of its various components. The detailed information of the ablation experiment is shown in Table 5.

The ablation study results demonstrate the progressive improvements achieved through successive model enhancements. Initial experiments using raw data input with the basic MCCNN+MAML architecture yielded a baseline accuracy of 84.64 percent across classification tasks. Subsequent implementation of ACMD preprocessing showed measurable accuracy gains, confirming the importance of signal decomposition for effective feature extraction. The most significant performance improvement occurred following ISENet module integration, with the complete MAML-MCCNN-ISENet framework achieving superior accuracy compared to SENet-based configurations at every shot level. This enhancement validates ISENet’s advanced capability in feature learning and adaptive weighting. Notably, the model showed consistent accuracy improvement with increasing sample size, as additional training data enables more comprehensive task understanding and better generalization. Comparative analysis revealed that while the Signal preprocessing+MCCNN+SENet+MAML configuration showed competent performance, the proposed method’s ISENet implementation consistently outperformed it across all experimental conditions. These results collectively demonstrate that optimal performance requires both proper signal preprocessing and advanced attention mechanisms, with sample quantity serving as an additional important factor in model effectiveness.

Ablation experiments show that the proposed method, which combines data preprocessing, ISENet module and MAML algorithm, shows excellent effect and potential in the problem of small samples.

#### 4.1.4. Comparative Experimental Analysis

**Figure 13:**
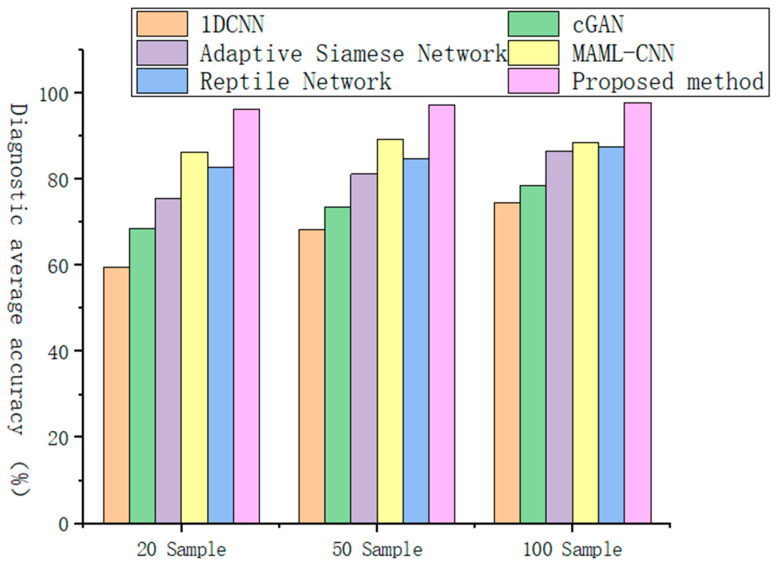
Accuracy of different tasks in different sample datasets on the laboratory plunger pump fault diagnosis dataset.

In order to verify the performance of the proposed method in the field of fault diagnosis, different comparative experiments were carried out in this study, and it was combined with the traditional 1DCNN Network, small sample learning algorithm (conditional generative adversarial networks) cGAN [30], Adaptive Siamese Network [31], and MAML-CNN. As well as existing methods such as Reptile network [32], performance comparisons were made on sample datasets of different sizes. The experimental evaluation employs three distinct sample sizes (20, 50, and 100 samples per operating condition) to thoroughly assess model robustness. As detailed in Table 6, our proposed method demonstrates superior performance across all test scenarios, achieving both the highest classification accuracy and most stable results (indicated by minimal standard deviation). Further supporting evidence appears in Figure 13, which visually compares task-specific accuracy distributions across different sample sizes, consistently showing our method’s performance advantage.

The experimental results demonstrate that the proposed MAML-MCCNN-ISENet framework consistently outperforms all baseline methods across varying sample sizes, maintaining superior accuracy levels throughout the evaluation. As the sample size increases, the model exhibits progressive performance enhancement, reliably achieving accuracy rates exceeding ninety percent regardless of the specific classification task configuration. Notably, while competing methods show expected accuracy improvements with additional samples, their performance remains consistently inferior to our model’s results—even our smallest-sample performance surpasses the best results achieved by alternative approaches using maximal sample sizes. This persistent performance advantage highlights the model’s exceptional few-shot learning capability and robust feature extraction architecture.

### 4.2. Case 2: Centrifugal Pump Vibration Dataset from the Saint-Longoval Institute of Engineering and Technology

#### 4.2.1. Experiment Description

**Figure 14:**
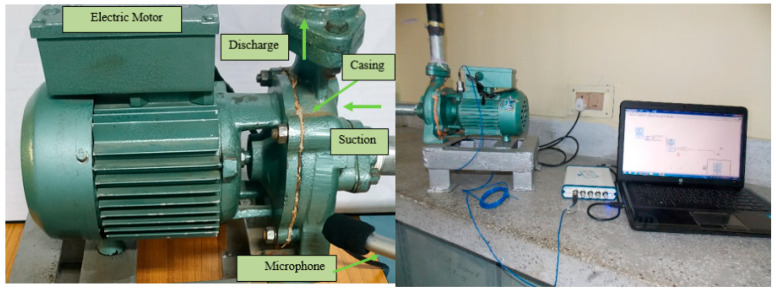
Centrifugal pump fault diagnosis test bench.

**Figure 15:**
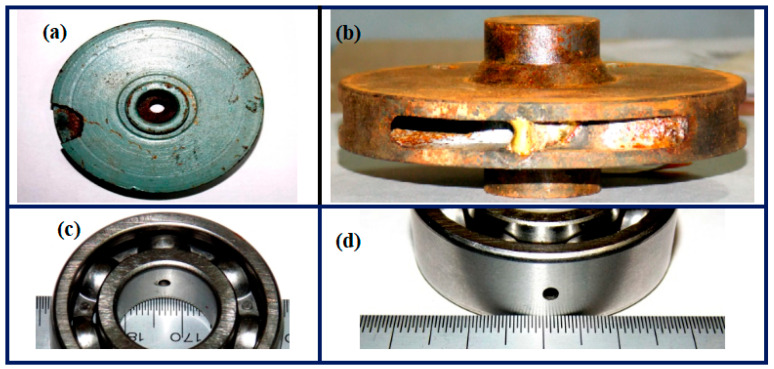
Four kinds of centrifugal pump faults: (**a**) impeller breakage, (**b**) impeller blockage, (**c**) bearing inner ring defect, (**d**) bearing outer ring defect.

To further validate the generalizability of our proposed method, we conducted additional experiments using the centrifugal pump vibration dataset from Saint-Longoval Institute of Engineering and Technology [33]. Figure 14 is the centrifugal pump fault diagnosis test bench. A total of five states of data were collected, namely normal, impeller fracture, impeller blockage, bearing inner ring defect and bearing outer ring defect. Four kinds of centrifugal pump faults are shown in Figure 15.

The experimental framework treats each of the five fault states as distinct classification categories, with the collected dataset systematically organized into labeled subsets corresponding to each fault type. For comprehensive evaluation, two independent sample groups are established per fault category, yielding ten representative sample sets in total. This complete dataset serves as the foundation for model training and validation, enabling the rigorous assessment of the method’s diagnostic accuracy and effectiveness for centrifugal pump fault detection.

Table 7 presents the configuration of the experimental dataset, comprising 20 uniformly selected sample instances per fault category. The evaluation protocol employs a rigorous temporal validation approach: initial model training utilizes sub-dataset A (containing earlier time-period data), while performance verification is conducted on temporally distinct sub-dataset B (collected during subsequent operation periods). This temporal segregation between training and testing datasets, while maintaining identical operational conditions, ensures a robust assessment of the model’s diagnostic capability and temporal generalizability.

#### 4.2.2. Analysis of Experimental Results

To rigorously evaluate the model’s diagnostic performance across diverse scenarios, we implemented a systematic experimental framework with multiple few-shot configurations (3-way 1/5/10/15-shot). In these experimental groups, each diagnostic task covered three separate types of centrifugal pump failure, whether during the training, validation, or testing phase. Critically, the support set for any given task contains a corresponding number of samples from each of the three fault types, for example, the support set for the 1-shot task has only one sample per fault class, while the 10-shot and 15-shot tasks provide 10 and 15 samples per fault class as support set data, respectively.

The query sets maintain symmetrical composition with their corresponding support sets, containing matching quantities of fault samples (1, 5, 10, or 15 per category) to ensure consistent evaluation of the model’s few-shot learning capability. Throughout the experimental process, we constructed 200 distinct 3-way multi-class fault diagnosis tasks, forming a comprehensive dataset that supports model parameter optimization during inner-loop iterations. This extensive task collection enables thorough evaluation of the model’s adaptive performance across diverse fault recognition scenarios.

Table 8 presents the comparative performance analysis of different methods across various few-shot configurations (3-way 1/5/10/15-shot). The results reveal two consistent patterns: first, all models exhibit progressive accuracy improvement with increasing sample size, and second, our proposed method consistently outperforms competing approaches, achieving a peak accuracy of 97.60% in the 15-shot condition. This performance advantage, particularly evident in data-scarce scenarios, confirms the model’s superior few-shot learning capability and robust generalization capacity for centrifugal pump fault diagnosis.

**Figure 16:**
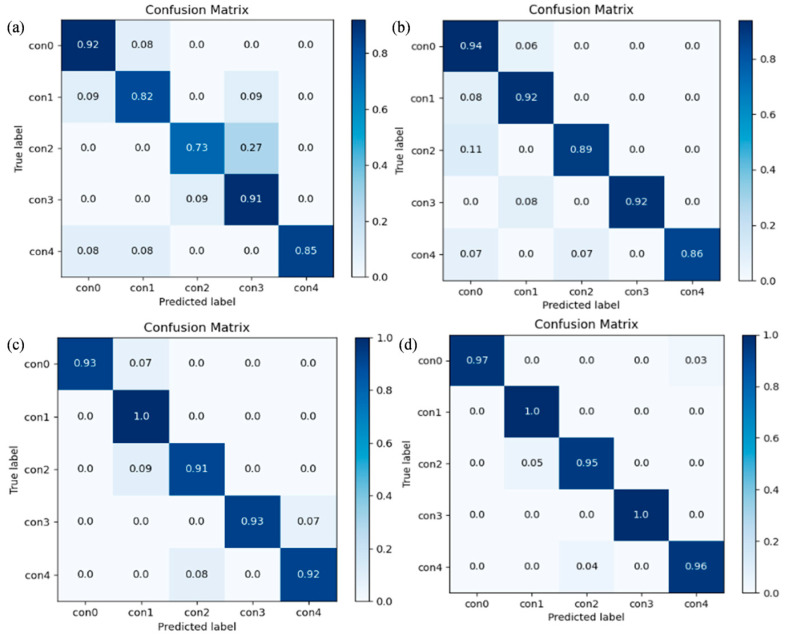
Confusion matrix of four classification tasks on the centrifugal pump vibration dataset (**a**) 3-way 1-shot task; (**b**) 3-way 5-shot task; (**c**) 3-way 10-shot task; (**d**) 3-way 15-shot task.

Figure 16 shows the specific classification results of the proposed model for test samples under different classification tasks in the form of confusion matrix. It can be seen that even the 1-shot classification task can maintain a recognition accuracy of more than 73% for all kinds of faults, while the 15-shot classification task can achieve a recognition accuracy of more than 95% for all faults. The overall average accuracy reaches 97.60%, which fully proves the high efficiency and reliability of the proposed model in the centrifugal pump fault diagnosis.

#### 4.2.3. Ablation Experiment Analysis

To systematically evaluate the contributions of individual model components, we conducted comprehensive ablation studies. Table 9 presents the detailed experimental results that quantitatively demonstrate the performance impact of each architectural element. These controlled experiments provide critical insights into the relative importance of different modules within our proposed framework.

Ablation experiment results show that the accuracy of each classification task is only 86% and 95% when the original data is directly input into MCCNN+MAML network for training. The second set of experiments uses data processed by ACMDWavelet threshold denoising, which shows that the accuracy is higher than that of the first set. This shows that preprocessing plays an active role in extracting effective features. Meanwhile, compared with the method presented in this paper, the third set of experiments showed that the model accuracy was significantly improved after the addition of ISENet module, which verified the effectiveness of ISENet in enhancing the feature learning and weight assignment ability of the model. The third group of experiments used the MAML-MCCNN-ISENet model and used the original data for training, and the accuracy of the model was reduced. At the same time, with the increase in the number of fault signals, the accuracy of various models is also improving. In the fourth group of experiments, in order to verify the effectiveness of ISENet, the Signal preprocessing+MCCNN+SENet+MAML model was trained using the original data, compared with the proposed method, the accuracy of ISENet is improved in each shot compared with SENet. The reason is that more training samples can provide more information, so that the model can learn the characteristics of the task more fully and make better generalization.

#### 4.2.4. Comparative Experimental Analysis

**Figure 17:**
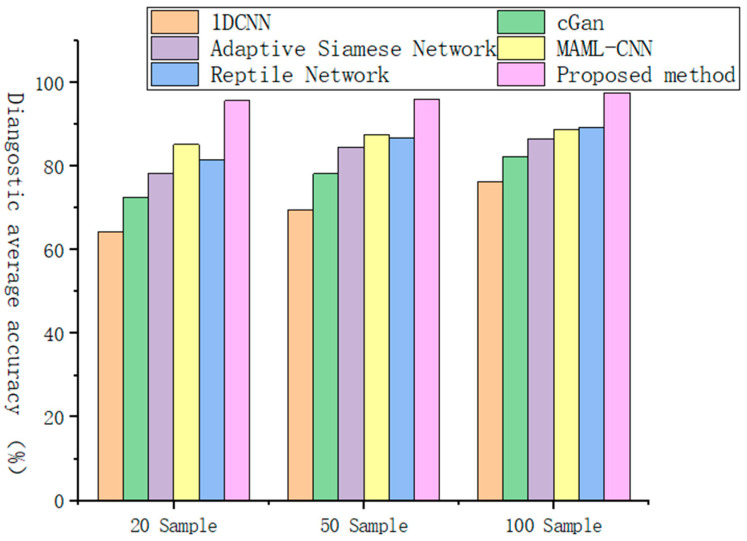
Accuracy of different tasks in different sample datasets on the centrifugal pump vibration dataset.

In order to verify the performance of the proposed method in the field of fault diagnosis, different comparative experiments were carried out in this study. Its performance was compared with existing methods such as the traditional 1DCNN Network, small sample learning algorithm cGAN [30], Adaptive Siamese Network [31], MAML-CNN, and Reptile network [32] on sample datasets of different sizes. The experimental evaluation employed three distinct sample sizes (20, 50, and 100 samples per operating condition) to systematically assess model performance. As detailed in Table 10, our proposed method demonstrates consistent superiority across all test scenarios, achieving both the highest classification accuracy and most stable results (as evidenced by minimal standard deviation values). These performance advantages are further corroborated by Figure 17, which visually compares task-specific accuracy distributions across the different sample sizes, uniformly showing our method’s leading performance position.

It can be seen that, compared with other comparison methods, the MAML-MCCNN-ISENet model proposed in this paper always maintains a higher accuracy level on datasets with different sample sizes, and with the increase in the number of samples, the accuracy of the model will also steadily improve. No matter what kind of sample size classification task, the accuracy of the model will be steadily improved and can achieve more than 90% accuracy.

### 4.3. Model Generalization Performance Verification

**Figure 18:**
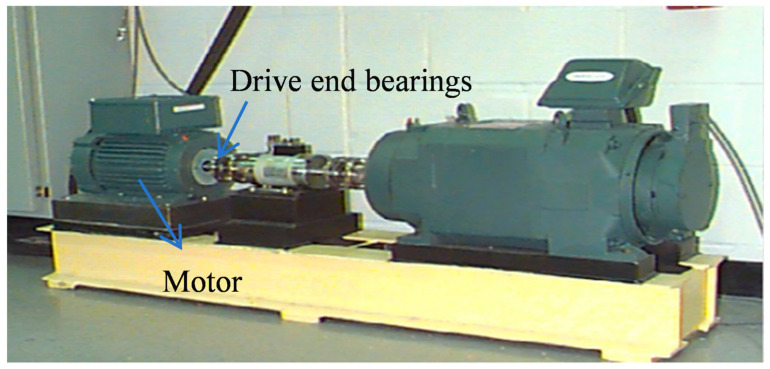
CWRU experimental platform.

To further validate the generalizability of our proposed method beyond pump components, we conducted additional experiments using the widely recognized Case Western Reserve University (CWRU) bearing dataset—a benchmark standard in fault diagnosis research. The experimental setup, as shown in Figure 18, provided drive-end bearing vibration data for comprehensive model evaluation, ensuring rigorous testing under established industry standards.

Table 11 shows the constructed dataset, 20 or 50 samples are selected for each dataset, the model is trained on dataset A, tested on dataset B, and a 3-way 15-shot classification task is set. The accuracy of the proposed method is 90.4% under 20 samples and 95% under 50 samples, both of which achieve high accuracy, which verifies the generalizability of the proposed method.

### 4.4. Discussion

In this paper, the proposed method is verified on the laboratory plunger pump dataset and the Saint-Longoval Institute of Engineering and Technology centrifugal pump dataset, the effectiveness of ISENet and MAML is proved by ablation experiments, and the other four small-sample learning methods are compared, the proposed method has the highest accuracy and the lowest standard deviation, and finally the generalization of the proposed method is verified on the bearing dataset of CWRU. The robustness of the model is not verified in this paper.

## 5. Conclusions

This paper proposes a fault diagnosis method of plunger pump based on meta-learning and improved multi-channel convolutional neural network (MAML-MCCNN-ISENet), which is used for fault diagnosis of plunger pump under small sample conditions and provides a new idea for intelligent fault diagnosis of plunger pump under small sample conditions. The MAML-MCCNN-ISENet model adds an improved SENet module to the multi-channel one-dimensional convolution to realize the adaptive setting of the signal feature weights of different channels by activating the dependency relationship between channels; the proposed MAML-MCCNN-ISENet model combined with the meta-learning strategy optimized the learning process of the model initialization parameters to solve the problem of insufficient generalization ability of the model in small sample scenarios.

In the actual industrial scenario, faults will gradually appear over time, so the dynamic update ability of the model is very important, and the combination of the proposed model and continuous learning can be considered in the future to realize the dynamic update of the model.

## Figures and Tables

**Table 1:** Network parameters.

Model Structure	Model Parameter	Output Channel
First convolution layer	Convolution kernel 64 × 1stride 16 padding 1, Maxpool kernel 2 × 1stride 2	16
First ISENet layer	Compression ratio 1/4	16
Second convolution layer	Convolution kernel 3 × stride1 × padding 1, Maxpool kernel 2 × 1stride 2	32
Second ISENet layer	Compression ratio 4	32
Third convolution layer	Convolution kernel 2 × stride1 × padding 1, Maxpool kernel 2 × 1stride 2	64
Third ISENet layer	Compression ratio 4	64
Fourth convolution layer	Convolution kernel 3 × stride1× padding 1, Maxpool kernel 2 × 1stride 2	64
Fourth ISENet layer	Compression ratio 4	64

**Table 2:** Information about the laboratory plunger pump fault diagnosis dataset.

Dataset Name	Direction of Rotation	Number of Operating Conditions	Sample Length	Sample Size
A	Forward	7	2048	7 × 20
B	Reverse	7	2048	7 × 20

**Table 3:** Model initialization parameters.

Parameter	Value
Internal learning rate	0.01
External learning rate	0.001
Number of Block	4
Batch_size	20
Inner ring optimizer	Adam
Outer ring optimizer	Adam
Loss function	CrossEntropyLoss
Training rounds	50
Data length	2048

**Table 4:** Accuracy rate of each classification task on the laboratory plunger pump fault diagnosis dataset.

Classification Task	Accuracy
3-way 1-shot	90.57
3-way 5-shot	93
3-way 10-shot	95
3-way 15-shot	97.29

**Table 5:** Ablation study results on the laboratory plunger pump fault diagnosis dataset.

Model	1-Shot	5-Shot	10-Shot	15-Shot
Original signal+MCCNN+MAML	81.28	84.28	84.47	84.64
Signal preprocessing+MCCNN+MAML	85.96	88.44	88.55	91.39
Original signal+MCCNN-ISENet+MAML	84.69	85.67	91.39	92.36
Signal preprocessing+MCCNN+SENet+MAML	87.23	89.48	91.69	94.21
Proposed method	90.57	93	95	97.29

**Table 6:** The results of diagnostic comparison experiment with different diagnostic models on the laboratory plunger pump fault diagnosis dataset.

Model	20 Sample	50 Sample	100 Sample
1DCNN	59.66 ± 1.23	68.34 ± 0.87	74.52 ± 0.65
cGAN	68.56 ± 1.34	73.61 ± 0.96	78.59 ± 0.81
Adaptive Siamese Network	75.62 ± 0.96	81.29 ± 0.74	86.59 ± 0.51
MAML-CNN (3-way 15-shot)	86.37 ± 0.41	89.25 ± 0.37	88.43 ± 0.24
Reptile network (3-way 15-shot)	82.79 ± 0.61	84.87 ± 0.53	87.64 ± 0.41
Proposed method (3-way 15-shot)	96.31 ± 0.21	97.28 ± 0.17	97.94 ± 0.09

**Table 7:** Information about the centrifugal pump vibration dataset.

Dataset Name	Number of Operating Conditions	Sample Length	Sample Size
A	5	2048	5 × 20
B	5	2048	5 × 20

**Table 8:** Accuracy rate of each classification task on the centrifugal pump vibration dataset.

Classification Task	Accuracy
3-way 1-shot	84.60
3-way 5-shot	90.60
3-way 10-shot	93.80
3-way 15-shot	97.60

**Table 9:** Ablation study results on the centrifugal pump vibration dataset.

Model	1-Shot	5-Shot	10-Shot	15-Shot
Original signal+MCCNN+MAML	79.62	83.54	85.68	86.95
Signal preprocessing+MCCNN+MAML	82.52	87.23	89.14	91.43
Original signal+MCCNN-ISENet+MAML	80.95	86.24	89.28	92.75
Signal preprocessing+MCCNN+SENet+MAML	83.06	88.12	91.97	95.12
Proposed method	84.60	90.60	93.80	97.60

**Table 10:** The results of diagnostic comparison experiment with different diagnostic models on the centrifugal pump vibration dataset.

Model	20 Sample	50 Sample	100 Sample
1DCNN	64.29 ± 0.86	69.58 ± 0.72	76.23 ± 0.64
cGAN	72.53 ± 0.99	78.25 ± 0.87	82.36 ± 0.69
Adaptive Siamese Network	78.37 ± 0.72	84.62 ± 0.61	86.57 ± 0.50
MAML-CNN (3way-15shot)	85.23 ± 0.46	87.61 ± 0.37	88.39 ± 0.35
Reptile network (3way-15shot)	81.58 ± 0.78	86.77 ± 0.61	89.23 ± 0.49
Proposed method (3way-15shot)	95.69 ± 0.29	96.17 ± 0.18	97.56 ± 0.08

**Table 11:** Construction of bearing dataset.

Rotate Speed/rpm	Load	Fault Diameter (Inches)	Fault Type/Label	Dataset Name
1730	1	0.007, 0.014, 0.021	Normal/0, Rolling element/1–3, Inner/4–6, Outer/7–9	A
1750	2	0.007, 0.014, 0.021		B

## Data Availability

The raw data supporting the conclusions of this article will be made available by the authors on request.

## Abbreviations

The following abbreviations are used in this manuscript:

ACMD	Adaptive Chirp Mode Decomposition
ISENet	Improved Squeeze and Excitation Networks
CNN	Convolutional neural network
SCAE	Stackable convolutional autoencoder
CWT	Continuous wavelet transform
BO	Bayesian optimization
VGG-LSTM	Visual Geometry Group-Long Short-Term Memory
MFAN	Multi-signal fusion adversarial
MAML	Model agnostic meta-learning
GMAML	Generalized model-agnostic meta-learning
WSMR	Weight space meta-representation
MWKN	Modified WaveletKernelNet
ADMTL	Attention-based deep meta transfer learning method
CAM	Channel attention mechanism
SENet	Squeeze and excite Network
GAP	Global average pooling
FC	Fully connected
